# Increased Expression of Immature Mannose-Containing Glycoproteins and Sialic Acid in Aged Mouse Brains

**DOI:** 10.3390/ijms20246118

**Published:** 2019-12-04

**Authors:** Frieder Simon, Kaya Bork, Vinayaga S. Gnanapragassam, Tim Baldensperger, Marcus A. Glomb, Simone Di Sanzo, Alessandro Ori, Rüdiger Horstkorte

**Affiliations:** 1 Institut für Physiologische Chemie, Martin-Luther-Universität Halle-Wittenberg, Hollystr. 1, D-06114 Halle/S., Germany; frieder.simon@uk-halle.de (F.S.); kaya.bork@medizin.uni-halle.de (K.B.); vinayaga.gnanapragassam@medizin.uni-halle.de (V.S.G.); 2 Institut für Chemie—Lebensmittelchemie, Martin-Luther-Universität Halle-Wittenberg, Kurt-Mothes-Str.2, D-06120 Halle/S., Germany; tim.baldensperger@chemie.uni-halle.de (T.B.); glomb@chemie.uni-halle.de (M.A.G.); 3 Leibniz Institute on Aging—Fritz Lipmann Institut, Beutenbergstr. 11, 07745 Jena, Germany; Simone.DiSanzo@leibniz-fli.de (S.D.S.); Alessandro.Ori@leibniz-fli.de (A.O.)

**Keywords:** sialic acid, neural cell adhesion molecule (NCAM), glycation, *O*-GlcNAc, mannose

## Abstract

Aging represents the accumulation of changes in an individual over time, encompassing physical, psychological, and social changes. Posttranslational modifications of proteins such as glycosylation, including sialylation or glycation, are proposed to be involved in this process, since they modulate a variety of molecular and cellular functions. In this study, we analyzed selected posttranslational modifications and the respective proteins on which they occur in young and old mouse brains. The expression of neural cell adhesion molecule (NCAM), receptor for advanced glycation endproducts (RAGE), as well as the carbohydrate-epitopes paucimannose and high-mannose, polysialic acid, and *O*-GlcNAc were examined. We demonstrated that mannose-containing glycans increased on glycoproteins in aged mouse brains and identified synapsin-1 as one major carrier of paucimannose in aged brains. In addition, we found an accumulation of so-called advanced glycation endproducts, which are generated by non-enzymatic reactions and interfere with protein function. Furthermore, we analyzed the expression of sialic acid and found also an increase during aging.

## 1. Introduction

**Figure 1:**
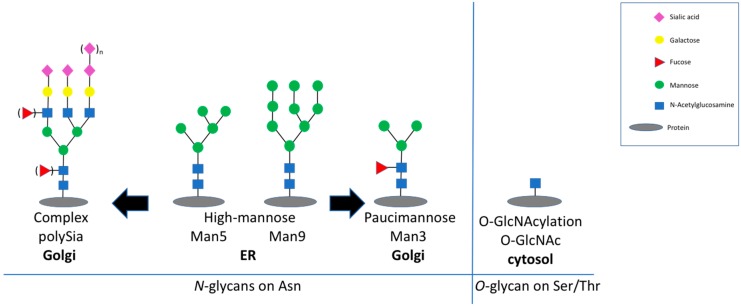
Structure of the analyzed glycans. On the left panel, some typical N-glycans are shown: High-mannose structures (From Man5 to Man9), which are assembled in the ER, whereas sialic acid-containing complex structures are generated in the Golgi. The right panel shows *O*-GlcNAc attached on serine or threonine residues in the cytosol by *O*-GlcNAc-transferase (OGT).

Aging is a physiological process resulting in a decline of several functions. The decline of function affects proteins, cells, and organs. One hallmark of molecular aging on protein level is related to posttranslational modifications. Two very prominent posttranslational modifications are glycosylation and glycation—both are related with carbohydrates. Glycosylation represents the attachment of glycans on proteins, which are then called glycoproteins. Proteins are glycosylated during their passage though the ER and the Golgi by a set of enzymes, which build specific glycan structures on serine/threonine (=O-glycan) or asparagine (=N-glycan) residues [1,2]. Glycans on glycoproteins are subdivided into high-mannose- or complex-type structures containing sialic acid (Figure 1). With the exception of albumin, most secreted and transmembrane proteins are glycoproteins. In general, glycosylation is important for the folding, the solubility, and the structure of the respective protein, and thereby modulates its function [3]. However, some glycan structures have more specific functions. One example is paucimannose (Figure 1), consisting of two N-acetylglucosamine, 1–3 mannose residues, and the option of a single fucose residue [4]. Paucimannose was shown to be involved in the differentiation of neural progenitor cells [5]. Another rare and interesting glycan of the brain is polysialic acid (polySia) [6]. PolySia is nearly exclusively expressed on the neural cell adhesion molecule NCAM (neural cell adhesion molecule). Expression of polySia interferes with the adhesion between cells by introducing negative charges and increasing the bound water [7,8]. PolySia is essential for normal brain development, but also during cancer progression of NCAM-expressing tumors [9]. However, there are many more functions of glycan structures, which cannot all be summarized in one single introduction. Disturbed glycosylation by genetic defects in the biosynthesis of glycan structures are called congenital disorders of glycosylation and lead to dramatic phenotypes, including mental retardation, indicating the importance of correct glycosylation for the function of the brain and other organs [10]. Attachment of a single N-acetylglucosamine (*O*-GlcNAc) to serine or threonine residues on intracellular proteins, so called *O*-GlcNAcylation, represent a dynamic glycosylation (Figure 1) [11]. Reversible *O*-GlcNAcylation opens the possibility to regulate protein or enzyme function similar to protein phosphorylation. However, little is known on the role of glycosylation during aging.

Glycation is a non-enzymatic chemical reaction between the carbonyl moiety of a reducing sugar or of dicarbonyls [12], such as methylglyoxal or glyoxal, which are derived during glycolysis or lipid peroxidation, and a free amino group of a protein. Further degradation of these products results in the formation of advanced glycation endproducts (AGEs) [13,14]. Formation of AGEs can lead to protein aggregation or misfunction, and accumulation of AGE-modified proteins is associated with the progression of several diseases (diabetes, Alzheimer’s disease, multiple sclerosis, or atherosclerosis) [15]. AGEs can also be recognized by some cellular receptors. The best characterized receptor for AGEs is the receptor for advanced glycation endproducts (RAGE) [16]. Binding of AGEs to RAGE leads to internalization and signal transduction [17], resulting in activation of p44/p42 mitogen-activated protein kinase and nuclear transcription factor κB (NF-κB) [18].

The aim of this study was to demonstrate that specific posttranslational modifications correlate with aging. We chose the brain, since aging is encompassed with a variety of changes, which are related to declined brain function. Therefore, we prepared protein fractions of brains of young (2 months) and old (22 months) mice and analyzed the expression of two mannose-rich structures (high-mannose and paucimannose), polySia, *O*-GlcNAc, and AGEs by Western blot. We found that the expression of mannose-containing paucimannose is highly upregulated during aging and that AGEs accumulate on a variety of proteins. One major paucimannose-carrying protein was identified as synapsin-1. 

## 2. Results

### Analysis of Glycans and Proteins by Western Blot

#### 2.1.1. PolySia-Expression Decreases with Age

**Figure 2:**
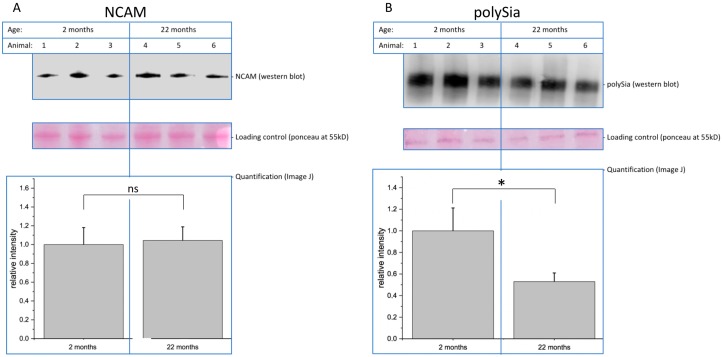
Brain membrane samples of 2-month-old and 22-month-old mice were separated by SDS-PAGE and analyzed by immunoblotting. (**A**) Neural cell adhesion molecule (NCAM) expression was detected using an anti-NCAM antibody (mab5B8) and quantified in relation to the loading control. Bars represent mean of relative NCAM expression + standard error of the mean (SEM) of three independent experiments (ns = not significant). (**B**) PolySia expression was detected using an anti-polySia antibody (mab735) and quantified in relation to the loading control. Bars represent mean of relative polySia-expression + SEM of three independent experiments (* *p* < 0.05).

In the first series of experiments, we quantified the expression of NCAM and polySia in young and aged mouse brains. Since polySia is known to be downregulated during development and aging, we used polySia as a positive control. Figure 2 shows the result of a representative Western blot analysis. The expression of polySia drops, as expected, by nearly 50% comparing brains of 2-month-old with 22-month-old mice. In contrast, the expression of the polySia carrier protein NCAM is unchanged and comparable between young and old mouse brains (Figure 2).

#### 2.1.2. Sialic Acid Expression Increases During Aging

**Figure 3:**
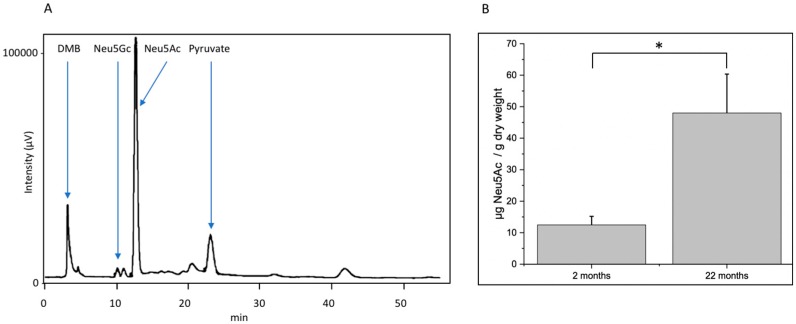
Sialic acids were hydrolyzed from brain membrane samples of 2-month-old and 22-month-old mice and labeled with DMB. Samples were analyzed by high performance liquid chromatography (HPLC). (**A**) Representative elution profile of one out of three HPLC runs of a 2-month-old animal (Neu5Ac = *N*-acetylneuraminic acid; Neu5Gc = *N*-glycolylneuraminic acid). (**B**) Quantification. Bars represent mean of relative sialic acid expression + SEM of three independent experiments (* *p* < 0.05).

Sialic acid (Sia) is the terminal component of all complex glycans and involved in a variety of functions. We quantified Sia after labeling with 1,2-diamino-4,5-methyleneoxybenzene (DMB) using high performance liquid chromatography (HPLC) analysis (Figure 3). Figure 3A shows a representative HPLC run, showing mainly three major peaks (Figure 3A): Uncoupled DMB, Sia (in this case, Neu5Ac = *N*-acetylneuraminic aicd, one major sialic acid), and pyruvate. We found that during aging (from 2 to 22 months), the expression of Sia increases dramatically from 12 µg to 48 µg per gram dry weight (Figure 3B).

#### 2.1.3. AGEs and the Receptor RAGE Accumulate During Aging in the Brain

**Figure 4:**
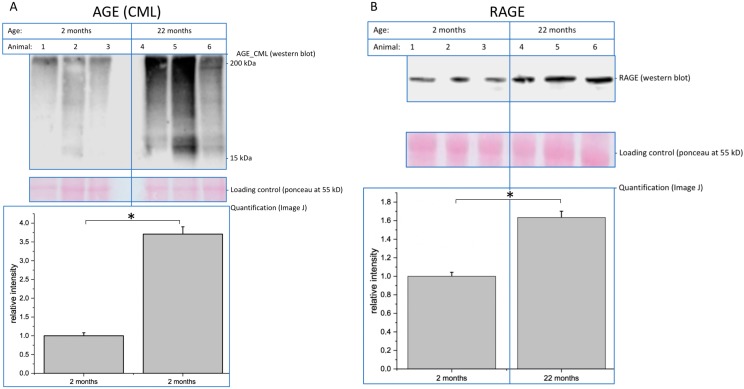
Brain membrane samples of 2-month-old and 22-month-old mice were separated by SDS-PAGE and analyzed by immunoblotting. (**A**) Expression of advanced glycation endproducts (AGEs) was detected using an anti-CML antibody and quantified in relation to the loading control. Bars represent mean of relative AGE expression + SEM of three independent experiments. (**B**) Receptor for advanced glycation endproducts (RAGE) expression was detected using an anti-RAGE antibody and quantified in relation to the loading control. Bars represent means of relative RAGE expression + SEM of three independent experiments (* *p* < 0.05).

It is known that glycated proteins, e.g., AGEs, are difficult to degrade by the cellular protein degradation machinery. In addition, accumulated proteins and protein aggregates are associated with neural disorders such as Alzheimer’s disease and also with reduced brain functions. Therefore, we quantified AGE-modified proteins from young and aged mouse brains using an anti-CML antibody. We could detect AGEs as a smear, indicating that many, if not most, proteins are AGE-modified in both young and old mice (Figure 4A). However, quantitative analysis via Western blot revealed more than 3 times more AGEs in brains of 22-month-old mice compared with 2-month-old mice (Figure 4A). It has been reported by several authors that increased expression of AGEs lead also to increased expression of the receptor for advanced glycation endproducts (RAGE). We therefore performed Western blot analysis using a monoclonal antibody to RAGE (Figure 4B). As expected, we found a 50% upregulation of RAGE in the brains of 22-month-old mice compared with 2-month-old mice.

#### 2.1.4. *O*-GlcNAc Expression Is Not Altered During Aging

In contrast to AGE modification, *O*-GlcNAcylation is a very dynamic posttranslational modification and is involved in the activation or deactivation of proteins or enzymes. Recently, it was suggested that *O*-GlcNAcylation is upregulated during aging in several organs [19]. Since *O*-GlcNAcylation occurs on many proteins, we separated water-insoluble proteins from soluble proteins by centrifugation. As expected, many proteins carried *O*-GlcNAc, and therefore, we detected many positive bands. However, after quantification of young and old samples, we could not find significant differences between 2- or 22-month-old mice, neither in the insoluble nor in the soluble fraction (Supplementary Figure S1).

#### 2.1.5. Mannose-Rich Glycoproteins Are Upregulated During Aging

**Figure 5:**
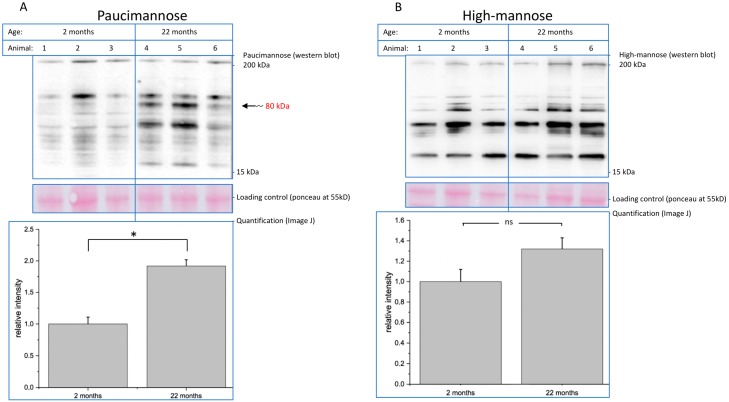
Brain membrane samples of 2-month-old and 22-month-old mice were separated by SDS-PAGE and analyzed by immunoblotting. (**A**) Paucimannose expression was detected using an anti-paucimannose antibody and quantified in relation to the loading control. Bars represent mean of relative paucimannose expression + SEM of three independent experiments (* *p* < 0.05). (**B**) High-mannose expression was detected using an anti-high-mannose antibody and quantified in relation to the loading control. Bars represent mean of relative high-mannose expression + SEM of three independent experiments (ns = not significant).

We next analyzed paucimannose- and high-mannose-containing glycoproteins by Western blot analysis (Figure 5). We detected many bands, since these epitopes are present on several glycoproteins. We quantified an increase of 92% of total paucimannose expression using the monoclonal Mannitou antibody (Figure 5A), and a moderate increase of 30% of total high-mannose structures using the monoclonal antibody 492 (Figure 5B). Note the staining in the 80 kDa range in the paucimannose blot, which is strongly increased in blots of aged mouse brain membrane proteins. We therefore immunoprecipitated paucimannose-containing structures from mouse brain membrane proteins, since *N*-glycosylation such as paucimannose is found only on membrane proteins.

**Figure 6:**
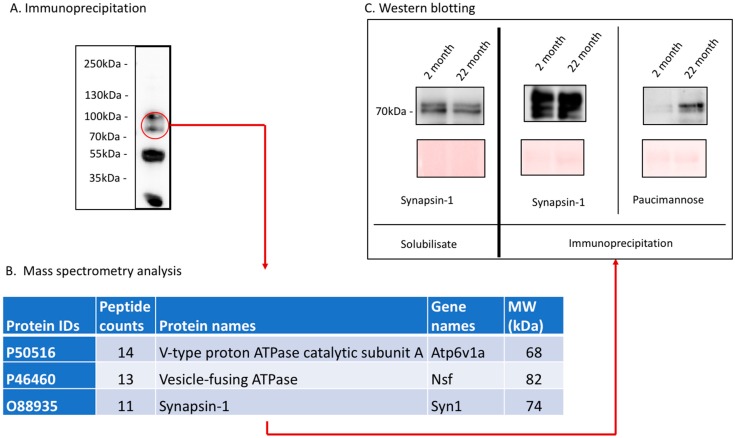
(**A**) Paucimannose expression was detected using an anti-paucimannose antibody. Corresponding region of a gel was cut out and proteins were analyzed by mass spectrometry. (**B**) Table of top three membrane proteins. The full list of proteins is provided in Supplementary Table S1. (**C**) Brain solubilizates of young and old mice were probed with anti-synapsin-1 antibodies or paucimannose antibodies before (left) and after (right) immunoprecipitation.

In Figure 6A, one representative blot of such a precipitation is shown. To identify the respective paucimannose-containing glycoprotein(s), we cut these bands out of the corresponding immune-precipitations and analyzed tryptic peptides by mass spectrometry. A list of the top three membrane proteins is shown in Figure 6B (the complete dataset is shown in Supplementary Table S1). To verify the data obtained by mass spectrometry, we performed additional immunoprecipitations using specific antibodies to these three top membrane proteins, and re-probed these with anti-paucimannose antibodies. We could not detect paucimannose on the precipitated vesicle fusing ATPase (data not shown) and had only a very weak signal on the precipitated C-type proton ATPase (data not shown) in both young and old mouse brains. The precipitated synapsin-1 of young mouse brains was hardly paucimannose positive, whereas the expression of paucimannose in aged mouse brains was strongly upregulated (Figure 6C). The signal for synapsin-1 itself was similar in both young and aged mouse brains (Figure 6C), indicating that only paucimannose on synapsin-1 and not synapsin-1 protein was upregulated during aging.

## 3. Discussion

Decreasing function of proteins is one hallmark of molecular aging. In this context, posttranslational modifications such as glycosylation play an important role. Here, we analyzed young (2-month-old) and aged (22-month-old) mouse brain tissue and found several interesting changes during aging. First, overall (poly)sialylation is reduced in aged mouse brains. This is of special interest, since sialylation, especially polysialylation, is involved in synaptic plasticity and, thereby, in learning and memory [20]. Although many authors reported that polysialylation is reduced during development and aging, it is not clear whether this decrease interferes with function or is a result of the cellular response to decreased function. We also found that total sialylation does not decrease during aging. In contrast, the amount of total sialic acid even slightly increases. However, polysialylation represents only a minor portion of all sialic acids, because only NCAM is polysialylated. Since all glycoproteins and the gangliosides are sialylated, the total sialylation overrules the effect of reduced polysialylation. The second interesting difference between young and aged mouse brains is the expression of mannose-containing epitopes. We analyzed this using specific monoclonal antibodies and found a moderate increase in total oligomannose structures. Oligomannosidic glycans are generated in the ER [21] and represent more immature glycans, since the glycosylation pathway starts with those structures before the trimming in the Golgi generates the complex glycosylation. The most striking observation we made was that, specifically, paucimannose (and not high-mannose) is strongly upregulated in aged mouse brains. Paucimannose represents a relatively simple structure containing N-acetylglucosamines and mannoses (see Figure 1). This epitope per se is involved in immune function [22] and has recently been shown to interfere with brain tumor progression [23]. Since paucimannose was strongly associated with specific molecular weights in Western blot analysis, we tried to identify carrier proteins of the upregulated paucimannose in aged brains. Using a combination of immunoprecipitation and mass spectrometry, we could identify synapsin-1 as one major carrier of paucimannose in aged mouse brains. Synapsin-1 is expressed on synaptic vesicles and involved in neurotransmitter release and synapse function [24]. Since synapse function is a key feature for neuronal function, the expression of paucimannose on synapsin-1 might be involved in the decreased function of aged brains.

Our data presented here were obtained from animal (mouse) brains, and we have to extend those investigations to human beings. Given that glycosylation is involved in human aging, even intervention might also be possible by supplementation of specific monosaccharides or precursors of those in the diet.

Finally, as a probe for AGEs, we found an expected increase in CML in aged brains. This is of special interest, since we previously demonstrated that glycation and the presence of advanced glycation endproducts interfere with neuronal function [25,26]. For example, neurite outgrowth, a marker of neuronal plasticity, is strongly reduced after cellular glycation. In addition, glycation of the extracellular matrix, as well as of neurotrophic factors, such as nerve growth factor, also interfere with neuronal function. Increased glycation leads to increased expression of the receptor for advanced glycation endproducts (RAGE). RAGE activation leads to pro-inflammatory signals in the respective cells [26]. We found increased RAGE expression in aged brains, which correlates with increased glycation. Higher expression of RAGE could result in increased pro-inflammatory signals in the central nervous system, which is known to interfere with its function.

Coming back to glycosylation and aging—several studies have investigated glycan patterns during aging using serum proteins or antibodies from humans as a source, and found, for example, decreased bisecting N-acetylglucosamine glycoforms on IgGs in elderly individuals [27]; or, for example, in the Leiden Longevity Study, non-sialylated di-antennary low sialylated structures in the offspring of long-lived individuals [28]. 

To summarize, we present a set of data demonstrating that a change in glycosylation and glycation occurs during aging, and that this might be one of several reasons for decreased brain function.

## 4. Materials and Methods 

### 4.1. Animals

For the extraction of the mice brains, three eight-week-old and three 22-month-old male BI6 mice were sacrificed (Janvier Labs, Le Genest Saint Isle, France). The animals were sacrificed and brains were taken out and stored at −80 °C. We had permission to use laboratory animals from the state Sachsen-Anhalt: LSA I2M10 (October 2011).

### 4.2. Homogenization of Mouse Brains

The brains were homogenized on ice using 4 mL of hypotonic homogenization buffer and a glass douncer. Proteinase-inhibitor cocktail (PIC 1:500) and phenylmethylsulfonyl fluoride (PMSF—1:1000) were added. To avoid tissue residues in the sample, the homogenization was repeated after centrifugation (1200 rpm, 4 °C, 15 min) with the same amount of buffer.

To separate insoluble and soluble cell constituents, the homogenate was divided into two parts and centrifuged for 40 min with 30,000× *g* at 4 °C. Per organ, two pellets containing mainly insoluble cell components and a supernatant containing mainly soluble cell components were received.

To test the separation of cell components, a Western blot was carried out using GAPDH as a marker for the soluble parts of the cell and histone H1 as a marker for the insoluble part.

### 4.3. Protein Solubilization 

To solubilize the pellet proteins, 500 µL RIPA buffer, as well as PIC and PMSF, were added. The mixture was stirred on ice using a magnetic stirrer for at least 90 min.

After centrifuging with 13,000 rpm at 4 °C for 20 min, the supernatant was taken and could be used to analyze the solubilized protein via SDS electrophoresis and Western blot. 

### 4.4. Precipitation of Soluble Protein 

The supernatant was mixed with cold acetone in the ratio of one to four to precipitate the protein. After 20 min of shaking, the precipitate was centrifuged down (13000 rpm, 20 min, 4 °C). The produced pellet could be solubilized in RIPA as described above. 

### 4.5. Delipidation of Pellet Fraction

To delipidate the pellet, 1 mL of chloroform/methanol (2:1) was added. After 20 min of shaking at room temperature, the mixture was centrifuged (20 min, 13,000 rpm, 4 °C). The supernatant containing lipids was then removed. 

### 4.6. Sialic Acid Hydrolysis 

After addition of 1 mL propionic acid (2 mol/L) to the delipidated pellet, the mixture was incubated for four hours at 80 °C under slight shaking. The supernatant was taken out after a 20 min centrifugation (13,000 rpm, 4 °C).

To remove the propionic acid, the samples were freeze-dried and then dissolved again in ultrapure water. For further purification of the sialic acid samples, an anionic exchange purification using Prefilled Poly-Prep^®^ columns (BIO-RAD Laboratories Inc., Hercules, CA, USA) were used (filled with AG^®^1, AG MP-1 and AG 2 Strong Anion exchange resin). Sialic acids were eluted from the column using 5 mL of propionic acid 1 mol/L. Again, the samples were freeze-dried to remove the acid and then dissolved in 500 µL of ultrapure water. 

### 4.7. DMB-Labeling of Sialic Acids

An equal volume of DMB-labeling agent was added to the sialic acid samples. After 2.5 h of incubation at 50 °C protected from light, the samples were centrifuged shortly to remove any potential particles. They were then fluorescently labeled and ready to be analyzed using the HPLC.

### 4.8. HPLC 

To measure the sialic acid concentration, 10 µL of labeled sample was injected into the device. A LiChroCART^®^250-4 LiChrospher^®^100 RP-18e (5 µm) column (Merck KGaA, Darmstadt, Germany) was used. The solvent consisted of a mixture of 86% water, 8% acetonitrile, and 6% methanol, and was used with a flow of 0.6 mL/min. The wavelength chosen for excitation was 373 nm, resulting in an emission at 448 nm. The concentrations were determined using a diluted standard of the according sialic acid (0.5–50 µmol/L, Thermo Fisher Scientific, Waltham, MA, US). The devices used are listed below: Jasco FP-2020 Plus Intelligent Fluorescence Detector, Jasco PU-2080 Plus Intelligent HPLC-Pump, Jasco LG-2080 Plus Quaternary Gradient Unit, Jasco DG-2080-54 4 Line Degasser, Jasco AS-2055 Plus Intelligent Sampler.

### 4.9. SDS-PAGE and Western Blot

For the SDS-PAGE, Novex™ WedgeWell™ 4–12% Tris-Glycine gels (Invitrogen by Thermo Fisher Scientific) were used. All gels were run under reducing conditions. Protein loading was 10 µg protein per lane. After gel electrophoresis, the separated proteins were blotted onto 9 × 7 cm nitrocellulose membranes (GE Healthcare, Little Chalfont, UK) in a blotting chamber (BIO-RAD Laboratories Inc., Hercules, CA, USA). After blotting, proteins were stained with ponceau staining solution (Carl Roth GmbH & Co. KG, Karlsruhe, Germany). Surplus ponceau was washed away with distilled water. The images were scanned and saved in jpeg format to use them as loading control. 

To avoid unspecific binding, the membrane had to be blocked for 60 min using 5% milk (Carl Roth GmbH & CO. KG, Karlsruhe, Germany) or 3% bovine serum albumin (Carl Roth GmbH & CO. KG, Karlsruhe, Germany) in TBS-T, depending on the antibody. The blocked membranes were washed three times with TBS-T. Afterwards, the membranes were incubated with the primary antibody in the appropriate dilution for 12 h at 4 °C. The membranes had to be washed again and then were incubated with the secondary antibody for 1 h at ambient temperature. After washing 4 times for 5 min with TBS-T, antibody binding was detected via chemiluminescence-coupled horseradish peroxidase-reaction using Immobilon^®^ Forte Western HRP Substrate (Millipore Corporation, Billerica, MA, USA). Images were taken using the Chemidoc XRS system (BIO-RAD Laboratories Inc., Hercules, CA, USA).

For quantification, band intensity of proteins of interest was transformed into numeric values using ImageJ (Wayne Rasband, National Institutes of Health, Bethesda, MD, USA) and normalized to the corresponding loading controls. All analyses and visualizations were performed using OriginPro 2018 software (OriginLab Corporation, Northampton, MA, USA). Paired Student’s *t*-test was used. Figures show the average mean + standard error of the mean (SEM), and levels of significance are represented within the figures.

### 4.10. Immunoprecipitation

First, 50 µL protein G-sepharose (SigmaAldrich, St. Louis, MO, USA) was precoated with 5 µg anti-Synapsin-1 overnight at 4 °C. Cell solubilizates were incubated for 2.5 h at 4 °C with sepharose beads, which were precoated protein G. Beads pelleted by centrifugation and prior to SDS-PAGE, pellets were washed twice with RIPA buffer and resuspended in 100 µL of sample buffer.

### 4.11. Antibodies

The following antibodies were used: Anti-Paucimannose (serum-free undiluted cell culture supernatant; clone: Mannitou, gift from B. Schmitz, Bonn, Germany); anti high-mannose (serum-free undiluted cell culture supernatant; clone 492, gift from B. Schmitz, Bonn, Germany); anti-polySia (1:10,000 dilution, clone 735 gift from R.Gerady-Schahn, Hannover Germany, IgG), anti-NCAM (1:1000 dilution clone 5B8 IgG Hybridoma Bank); anti-CML (1:10,000 dilution; clone CML56 abcam); anti-O-GlcNAc (1:10,000 dilution; clone CTD110.6, Cell Signaling); anti-Synapsin-1 (1:5000 dilution; polyclonal 64581 Abcam); anti-RAGE (1:1000 dilution; polyclonal 3611 Abcam).

### 4.12. Bicinchoninic Acid Assays

Protein concentrations were measured using the BCA-protein assay kit (Thermo Fisher Scientific, Waltham, MA, US). The samples were diluted 1:10 in ultrapure water. A volume of 10 µL per sample was pipetted into a single well of a 96-well plate. After the addition of 200 µL of BCA solution per well, the samples were incubated for 30 min at 37 °C. Diluted bovine serum albumin (Carl Roth GmbH & CO. KG, Karlsruhe, Germany) was used as concentration standard. Absorption values were measured at 560 nm using a plate reader (Thermo Fisher Scientific, St. Louis, MO, USA).

### 4.13. Sample Preparation for Mass Spectrometry

The bands from the Coomassie-stained SDS-PAGE gels were excised, reduced in DTT (10 mM and 100 mM Ambic, 56 °C, 30 min), and subsequently alkylated with iodacetamide (55 mM and 100 mM Ambic, 25 °C, 20 min in the dark). Following dehydration with acetonitrile, trypsin (1 ng/μL solution in 50 mM ammonium bicarbonate) was added and the gel pieces were allowed to swell on ice for 30 min. They were then digested overnight at 37 °C with shaking. After digestion, the peptide content was extracted twice with sonication (using a solution of 50:50 water/acetonitrile, 1% formic acid). The pooled extracts were placed in a clean tube and dried with a speed vacuum centrifuge. The dried pool was finally re-dissolved in 50 μL of Oasis Solvent A (water, 0.05% formic acid). The digests were then desalted with Waters Oasis^®^ HLB µElution Plate 30 µm in the presence of a slow vacuum, following the manufacturer’s instructions. The eluates were dried down with the speed vacuum centrifuge and dissolved in 20 µL 5% acetonitrile, 95% Milli-Q water, with 0.1% formic acid prior to analysis by LC–MS/MS.

### 4.14. LC–MS/MS

Peptides were analyzed by tandem mass spectrometry (LC–MS/MS), as described [29]. In brief, peptides were separated using the nanoAcquity UPLC system (Waters) fitted with a trapping (nanoAcquity Symmetry C_18_, 5 µm, 180 µm × 20 mm) and an analytical column (nanoAcquity BEH C_18_, 1.7 µm, 75 µm × 250 mm). The outlet of the analytical column was coupled directly to an Orbitrap Fusion Lumos (Thermo Fisher Scientific) using the Proxeon nanospray source. Solvent A was water, 0.1% formic acid, and solvent B was acetonitrile, 0.1% formic acid. The samples (1 µL) were loaded with a constant flow of solvent A (5 µL/min) onto the trapping column. Trapping time was 6 min. Peptides were eluted via the analytical column with constant flow (0.3 µL/min). During the elution step, the percentage of solvent B increased in a linear fashion from 3 to 2% in 30 min, then increased to 32% in 5 more minutes, and finally to 50% in a further 0.1 min. Total runtime was 60 min. The peptides were introduced into the mass spectrometer via a Pico-Tip Emitter 360 µm OD × 20 µm ID; 10 µm tip (New Objective) and a spray voltage of 2.2 kV was applied. The capillary temperature was set at 300 °C. The RF lens was set to 30%. Full scan MS spectra with mass range 375–1500 *m/z* were acquired in profile mode in the Orbitrap with resolution of 120,000. The filling time was set at maximum of 50 ms with limitation of 2 × 10^5^ ions. The “Top Speed” method was employed to take the maximum number of precursor ions (with an intensity threshold of 5 × 10^3^) from the full scan MS for fragmentation (using HCD collision energy, 30%) and quadrupole isolation (1.4 Da window) and measurement in the ion trap, with a cycle time of 3 s. The MIPS (monoisotopic precursor selection) peptide algorithm was employed, but with relaxed restrictions when too few precursors meeting the criteria were found. The fragmentation was performed after accumulation of 2 × 10^3^ ions or after filling time of 300 ms for each precursor ion (whichever occurred first). MS/MS data were acquired in centroid mode, with the Rapid scan rate and a fixed first mass of 120 *m/z*. Only multiply charged (2^+^ – 7^+^) precursor ions were selected for MS/MS. Dynamic exclusion was employed with maximum retention period of 60 s and relative mass window of 10 ppm. Isotopes were excluded. Additionally, only one data dependent scan was performed per precursor (only the most intense charge state selected). Ions were injected for all available parallelizable time. In order to improve the mass accuracy, a lock mass correction using a background ion (*m/z* 445.12003) was applied. For data acquisition and processing of the raw data, Xcalibur 4.0 (Thermo Scientific) was employed.

### 4.15. Data Analysis 

For the qualitative label-free analysis, raw files from the Orbitrap Fusion Lumos were analyzed using MaxQuant (version 1.5.3.28) [30]. MS/MS spectra were searched against the *Mouse* Swiss-Prot entries of the Uniprot KB (database release 2016_01, 16,756 entries) using the Andromeda search engine [31]. A list of common contaminants was appended to the database search. The search criteria were set as follows: Full tryptic specificity was required (cleavage after lysine or arginine residues, unless followed by proline); three missed cleavages were allowed; oxidation (M) and acetylation (protein N-term) were applied as variable modifications, with mass tolerances of 20 ppm set for precursor and 0.5 Da for fragments. The reversed sequences of the target database were used as a decoy database. Peptide and protein hits were filtered at a false discovery rate of 1% using a target-decoy strategy [32]. The unique peptides and iBAQ values per protein (from the proteinGroups.txt output of MaxQuant) were used for further analysis. Only proteins with a minimum of two peptides per protein were retained and the top 10 per sample reported (based on unique peptide number).

## Supplementary Materials

Supplementary materials can be found at https://www.mdpi.com/1422-0067/20/24/6118/s1.

## Abbreviations

AGE	Advanced glycation endproducts
Man	Mannose
HPLC	High performance liquid chromatography
Sia	Sialic acid
