# Supplementary material for: Increased Expression of Immature Mannose-Containing Glycoproteins and Sialic Acid in Aged Mouse Brains
Source: Int J Mol Sci. 2019 Dec 4;20(24):6118. doi: 10.3390/ijms20246118 (PMC6940728; doi:10.3390/ijms20246118)
Supplement: Supplementary file 1 [file ijms-20-06118-s001.zip › ijms-637365 suppl for final/ijms-637365 suppl fig for final.pdf]

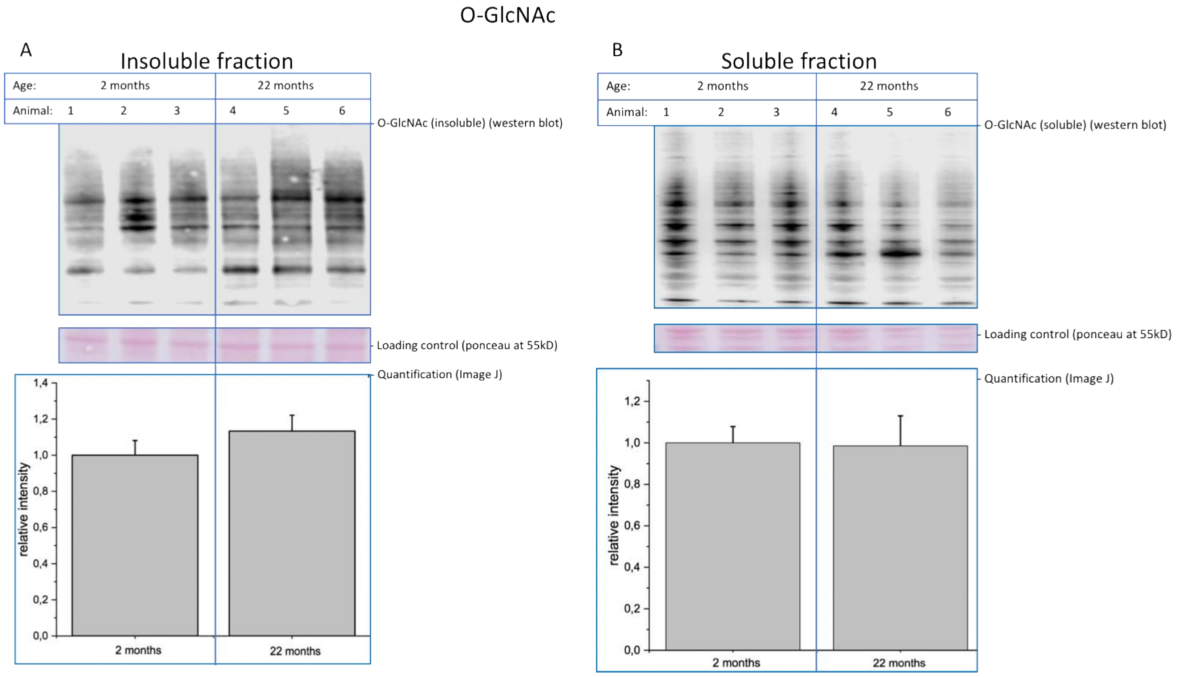

Supplementary Figure S1. Brain samples of 2-month-old and 22-month-old mice were separated by centrifugation into an insoluble (**A**) or soluble (**B**) fraction. Both fractions were subjected to SDS-PAGE and analyzed by immunoblotting. O-GlcNAc-expression was detected using an O-GlcNAc antibody and quantified in relation to the loading control. Bars represent mean of relative O-GlcNAc expression + SEM of three independent experiments.
